# Substance of ‘phantom’ cardiac masses confirmed by therapeutic outcome

**DOI:** 10.1007/s12471-021-01587-2

**Published:** 2021-06-02

**Authors:** D. Afendoulis, N. Papagiannis, M. Moutafi, G. Panagi, P. Voutas, S. Garoufalis, N. Smyrnioudis, A. Kartalis

**Affiliations:** 1 Department of Cardiology, General Hospital of Chios, Chios, Greece; 2 Department of Radiology, General Hospital of Chios, Chios, Greece

An 87-year-old woman presented to the emergency department of our hospital with acute-onset chest pain and a presyncopal episode. She was haemodynamically stable with sinus tachycardia and 93% oxygen saturation. Urgent transthoracic echocardiography and transoesophageal echocardiography (Fig. 1; Movie 1, Electronic Supplementary Material) demonstrated enormous, highly mobile masses in both atria, protruding into both ventricles, an intact interatrial septum, mild systolic right ventricular dysfunction and pulmonary hypertension. CT pulmonary angiography (CTPA) displayed the atrial masses and bilateral pulmonary embolism at the main pulmonary artery branches (Fig. 2a, b). Considering that our patient was at high risk of bleeding from thrombolysis [1–3] and the substance of the masses was unknown, low-molecular-weight heparin was initiated. Duplex ultrasonography of the lower extremities and total-body computed tomography displayed no signs of deep vein thrombosis or active malignancy. One week later she was discharged in a stable condition on direct oral anticoagulants. Follow-up echocardiography and CTPA demonstrated complete resolution of the cardiac masses, confirming the diagnosis of ‘heart thrombi’.

**Fig. 1:**
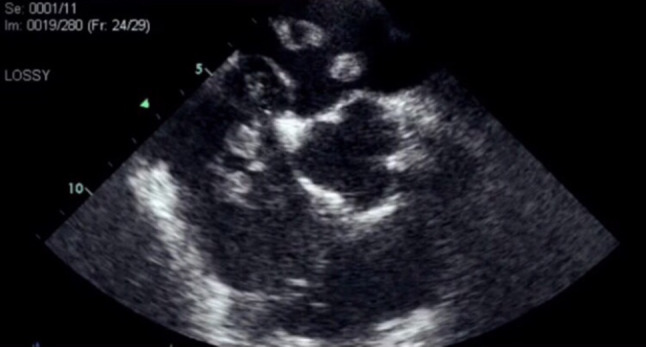
Transoesophageal echocardiogram, short axis view, displaying the masses within both atria

**Fig. 2:**
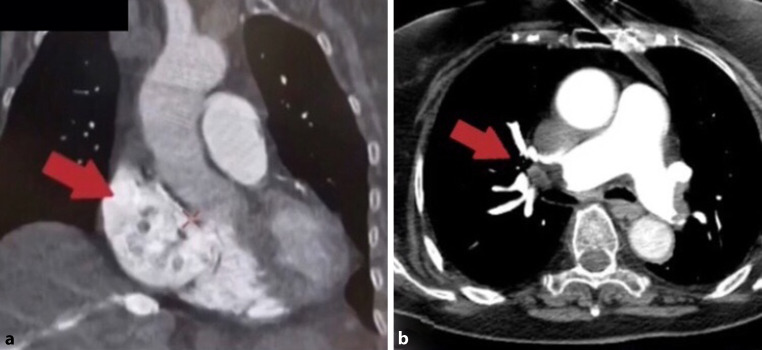
**a** CT pulmonary angiography (CTPA), sagittal view, depicting the presence of masses within the right atrium (*red arrow*). **b** CTPA, axial view, depicting bilateral pulmonary embolism (*red arrow*)

## Supplementary Information

Urgent transthoracic and transesophageal echocardiogram depicting enormous, mobile masses in the cardiac chambers
